# Recent-onset atrial fibrillation: a study exploring the elements of Virchow’s triad after cardioversion

**DOI:** 10.1007/s10840-021-01078-9

**Published:** 2021-10-24

**Authors:** Panagiotis Arvanitis, Anna-Karin Johansson, Mats Frick, Helena Malmborg, Spyridon Gerovasileiou, Elna-Marie Larsson, Carina Blomström-Lundqvist

**Affiliations:** 1 Department of Medical Science and Cardiology, Uppsala University, Sjukhusvägen 9, Ing 35, 75309 Uppsala, Sweden; 2 Department of Clinical Science and Education, Division of Cardiology, Karolinska Institutet, South Hospital, Stockholm, Sweden; 3 Department of Medical Sciences, Uppsala University, Clinical Physiology and Cardiology, Uppsala University, Uppsala, Sweden; 4 Department of Surgical Science, Radiology, Uppsala University, Uppsala, Sweden

**Keywords:** Recent-onset atrial fibrillation, Left atrial remodeling, Inflammation and coagulation systems, White matter hyperintensities

## Abstract

**Purpose:**

Atrial fibrillation (AF) imposes an inherent risk for stroke and silent cerebral emboli, partly related to left atrial (LA) remodeling and activation of inflammatory and coagulation systems. The aim was to explore the effects of cardioversion (CV) and short-lasting AF on left atrial hemodynamics, inflammatory, coagulative and cardiac biomarkers, and the association between LA functional recovery and the presence of a prior history of AF.

**Methods:**

Patients referred for CV within 48 h after AF onset were prospectively included. Echocardiography and blood sampling were performed immediately prior, 1–3 h after, and at 7–10 days after CV. The presence of chronic white matter hyperintensities (WMH) on magnetic resonance imaging was related to biomarker levels.

**Results:**

Forty-three patients (84% males), aged 55±9.6 years, with median CHA_2_DS_2_-VASc score 1 (IQR 0–1) were included. The LA emptying fraction (LAEF), LA peak longitudinal strain during reservoir, conduit, and contractile phases improved significantly after CV. Only LAEF normalized within 10 days. Interleukin-6, high-sensitivity cardiac-troponin-T (hs-cTNT), N-terminal-pro-brain-natriuretic peptide, prothrombin-fragment 1+2 (PTf1+2), and fibrinogen decreased significantly after CV. There was a trend towards higher C-reactive protein, hs-cTNT, and PTf1+2 levels in patients with WMH (*n*=21) compared to those without (*n*=22). At 7–10 days, the LAEF was significantly lower in patients with a prior history of AF versus those without.

**Conclusion:**

Although LA stunning resolved within 10 days, LAEF remained significantly lower in patients with a prior history of AF versus those without. Inflammatory and coagulative biomarkers were higher before CV, but subsided after 7–10 days, which altogether might suggest an enhanced thrombogenicity, even in these low-risk patients.

**Supplementary Information:**

The online version contains supplementary material available at 10.1007/s10840-021-01078-9.

## Introduction

The mechanism of thrombogenesis is complex and multifactorial and as described by Rudolf Virchow over a century ago, there are three main elements: (i) hemodynamic changes with abnormal blood flow, (ii) hypercoagulability, and (iii) endothelial dysfunction [1, 2]. Atrial fibrillation (AF) carries an inherent risk for intracavitary thrombus formation and with time electrical, structural, and functional remodeling of the left atrium (LA) result in atrial mechanical stunning [3–5]. Cardiomyocyte apoptosis, endothelial injury, and replacement fibrosis attribute to irreversible remodeling, leading to LA dilatation and dysfunction [6]. The functional recovery, following restoration of sinus rhythm, depends on the extent of irreversible structural remodeling and is thought to contribute to the development of silent or clinically evident cerebrovascular thromboembolic events, as the majority occur within 10 days after restoration of sinus rhythm [4, 5, 7–10]. Virchow’s triad of thrombogenicity is completed by the activation of inflammatory and coagulative system during AF. Although the activation of these systems may potentially explain the high incidence of silent, chronic white matter hyperintensities (WMH) observed on magnetic resonance imaging (MRI) in AF patients with AF, it remains unclear whether microemboli, direct microvascular injury, or transient cerebral hypoperfusion play a role in the pathophysiologic mechanisms of WMH [11–14].

The aim of the study was to assess the effects of a short-lasting AF episode (<48 h) and electrical cardioversion (CV) on thrombogenicity in anticoagulant-naïve patients by analyzing LA hemodynamics and biomarkers reflecting hypercoagulability on various levels, furthermore to explore whether a prior history of AF affects the degree of atrial functional recovery.

## Methods

### Study population

Patients with recent onset of AF within 48 h, naïve to oral anticoagulant and eligible for rhythm control treatment with CV, were prospectively included in the study; the details of which have been described previously [15]. In brief, patients were recruited from the emergency departments of two university hospitals in Sweden. Exclusion criteria were known moderate to severe valvular heart disease, previous or acute cerebrovascular event or thromboembolism, moderate or severe left ventricular systolic dysfunction, known coagulation defects, and contraindications for MRI and CV for AF in the preceding 3 months. Electrical CV was performed without heparin or low molecular weight heparin periprocedurally. The study was approved by the Regional Ethical Review Board and complied with the Declaration of Helsinki. Written informed consent was obtained from each study participant.

### Endpoints

The primary objective was to assess the effects of a short-lasting AF episode and CV on LA size, function, and myocardial deformation by echocardiography as well as on the inflammatory, coagulative, and cardiac/endothelial systems, by measuring corresponding biomarkers in anticoagulant-naïve patients with recent-onset AF and further to explore the association between the degree of atrial functional recovery and the presence of prior history of AF. The finding of WMH on brain MRI, as described in our previous study, urged us to further explore its relation to coagulative and inflammatory biomarkers [15].

**Fig. 1:**
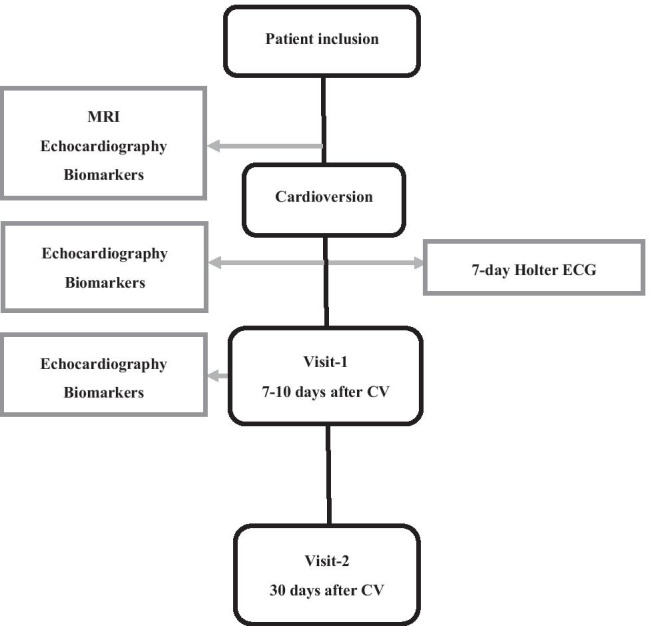
Study flowchart. CV: cardioversion; ECG: electrocardiography; MRI: magnetic resonance imaging.

In order to cover the time intervals associated with LA reverse remodeling and functional recovery, the echocardiographic examinations and blood sampling were scheduled within 2 h prior to CV, 1–3 hours after, and at 7–10 days after CV, representing the recovery period of the LA contractile function with the highest thromboembolic events [4, 5, 8, 15]. The last follow-up was at 30 days after CV (Fig. 1).

### Echocardiographic assessment

Transthoracic echocardiography was performed using General Electric Vivid E9 ultrasound system and according to recommendations by the European Association of Cardiovascular Imaging [16, 17]. Left atrial volumes were measured using the modified Simpson’s method at the apical four-chamber view. The LA maximal end-diastolic volume (LAEDV) was measured at the beginning of cardiac diastole, the end-systolic volume (LAESV) at the end of cardiac diastole, and the total emptying fraction (LAEF) was calculated as (LAEDV−LAESV)/LAEDV × 100. The LA volume indexed to body surface area (LAVI) was also calculated. Speckle tracking echocardiography (STE) for LA deformation analysis was assessed offline using General Electric EchoPAC software version 201. The LA peak longitudinal strain during reservoir phase at the end of ventricular diastole until mitral valve opening (LAεR), during conduit phase until the onset of atrial contraction (LAεCD), and during contraction phase until the end of the ventricular diastole (LAεCT) were measured. The zero strain reference was set at left ventricular end-diastole. Left ventricular volumes were measured at the end of left ventricular diastole (LVEDV) and systole (LVESV) using the modified Simpson’s method and left ventricular ejection fraction (LVEF) was calculated as (LVEDV−LVESV)/LVEDV×100. Echocardiographic variables were compared in patients with and without the presence of a history of paroxysmal or persistent AF. All measurements were made by one physician experienced in echocardiographic imaging and blinded to patient demography. The values were the mean of 3 consecutive measurements.

### Magnetic resonance imaging

The brain MRI was performed prior to CV as described earlier [15]. In brief, chronic deep white matter lesions or white matter hyperintensities were visually quantified using the Fazekas scale where 0 equals normal, 1 is assigned to punctate lesions, and 3 is assigned to the most extreme cases where extensive and confluent lesions are present [18]. Patients with and without WMH were compared with regard to levels of inflammatory and coagulative biomarkers.

### Biomarkers

Blood sampling for biomarkers was performed 2 h prior to CV, 1–3 h, and 7–10 days after CV. Inflammatory biomarkers included interleukin-6 (IL-6), C-reactive protein (CRP), and P-selectin. Cardiac biomarkers included high-sensitivity cardiac troponin T (hs-cTNT) and N-terminal pro-brain natriuretic peptide (NT-proBNP). Coagulation biomarkers included prothrombin fragment 1+2 (PTf1+2), von Willebrand Factor antigen (vWFag), coagulation factor VIII, fibrinogen, and D-dimer. Specific methods for analyzing biomarkers are presented in *supplement table* *1*.

### Statistical analysis

The variables were tested for normality using Shapiro-Wilk test. The results were reported as mean ± standard deviation (SD) for normally distributed variables, as median (interquartile range) for non-normally distributed variables and counts, *n*, and percentage (%) for the remaining. Normally distributed variables were analyzed using Student *t* test for pairwise comparison and repeated measures analysis of variance (rmANOVA) with the use of Greenhouse-Geisser or Huynh-Feldt correction for violation of Mauchly’s test of sphericity for serial results analysis. Variables with a non-normal distribution were analyzed using Wilcoxon signed-rank test for their pairwise comparisons, adjusting significance values by the Bonferroni correction for multiple tests, and Friedman’s two-way analysis of variance by ranks for serial result analysis. A value of *p* < 0.05 was considered significant. Missing values classified as “missing at random” were treated with single imputation [19]. Left-censored data below the lower limit of quantification (LOQ) were treated with simple substitution with LOQ/√2. Repeatability analysis was performed on 42 echocardiographic examinations by two raters (SG and PA), at two different time points, using the intraclass correlation coefficient (ICC). A two-way random-effect model based on average ratings and absolute agreement assessed the inter-rater repeatability. Mean estimations along with 95% confidence intervals (CI) were reported for each ICC; interpretation was as follows: <0.50, poor; between 0.50 and 0.75, fair; between 0.75 and 0.90, good; above 0.90, excellent. All statistics were analyzed with IBM Statistical Package for the Social Sciences, version 27 for Windows (IBM Corp. Released 2017. IBM SPSS Statistics for Windows, Armonk, NY).

## Results

### Study population

Forty-three patients were included, of whom 26/43 (60.5%) had a prior history of paroxysmal or persistent AF (Table 1). The CHA_2_DS_2_-VASc score [20] was ≤1 in 40 (93.8%) patients. Six patients (6/43) had undergone CV within twelve months (4–8.8 months) prior to inclusion; two of which have had persistent AF.

**Table 1:** Basic characteristics of study population

Variables	Patients *n* = 43
Gender, males	36 (83.7)
Age, yrs.	55±9.6
BMI, kg/m^2^	26±3.4
Hypertension	9 (20.9)
Diabetes Mellitus	2 (4.7)
Vascular disease	1 (2.3)
Systolic BP (mmHg)	125±13
Diastolic BP (mmHg)	81±9
Plasma Creatinine, μmol/L	82.8±13.5
CHA_2_DS_2_-VASc = 0	21 (48.8)
CHA_2_DS_2_-VASc = 1	19 (44.2)
CHA_2_DS_2_-VASc ≥ 2	3 (6.9)
First-onset AF	17 (39.5)
Previous history of paroxysmal AF	17 (39.5)
Previous history of persistent AF	9 (20)
AF duration from symptom onset to diagnosis, hours	13.3±11.7
Heart rate at presentation (bpm)	110±31
ACE/ARB medication	8 (18.6)
Antiplatelet therapy	0 (0)
Beta receptor blockers	9 (20.9)

Figures are mean ± one standard deviation or frequencies and percentages; *ACE*, angiotensin-converting enzyme; *AF*, atrial fibrillation; *ARB*, aldosterone receptor blocker; *BMI*, body mass index; *BP*, blood pressure; *CV*, cardioversion; *Kg*, kilogram; *m*, meter; *ml*, milliliter; *yrs.*, years

Forty-one patients were successfully cardioverted while 2 patients converted spontaneously to sinus rhythm after the first MRI examination. The 7-day Holter monitoring after CV-detected AF recurrence in 4/43 (9.3%) patients of whom 3 had paroxysmal AF with durations 33–183 s and one had persistent AF scheduled for repeat elective CV. Seven of the 43 (16.2%) patients were initiated on novel oral anticoagulants after CV and continued medication during the study period; 3 of these patients had CHA_2_DS_2_-VASc score ≥2 and 4 patients had score 1. Another patient received oral anticoagulants on the 6th day due to peripheral emboli related to Leiden mutation.

Missing data, categorized as randomly missing, reached 14.5%.

### Echocardiographic assessment

The baseline LAVI was normal in all patients. There were no significant differences in the serial LAVI measurements prior CV, 1–3 h after and at 7–10 days after CV using rmANOVA. There was a statistically significant overall decrease in LAESV from prior CV, 1–3 h after CV to the 7–10 days after CV. Consequently, there was a corresponding statistically significant overall improvement in LAEF, from prior CV, 1–3 h after CV to 7–10 days after CV using rmANOVA, which remained stable for both patients with and without prior history of AF (Table 2). Although the LAEF normalized at 7–10 days, LAEF at 7–10 days was significantly lower in those with versus those without a prior AF history, 50.3±8.8 versus 56.3±8.7, respectively, *p*=0.032. A statistically significant improvement in all three LA deformation indices, LAεR, LAεCD, and LAεCT, for the entire cohort, and a trend of lower values at baseline were observed in those with a prior AF history. Moreover, when comparing LA function after restoration of sinus rhythm at 1–3 h versus 7–10 days, there was a statistically significant increase of the LAEF t(42)=−2.4 *p*=0.023, LAεR *t*(42)= −4.4 *p*<0.001, LAεCD *t*(42)= −2.7 *p*=0.009, and LAεCT *t*(42)= −4.2 *p*<0.001.

**Table 2:** Echocardiographic variables before and after electrical cardioversion

Echocardiographic parameter	Prior to CV	After CV	7–10 days after CV	Repeated measure ANOVA	*p* value
LAVI (mL/m^2^)	31.3±9.4	32.3±9.3	31.8±10.9	*F*(1.89 , 76.4)=0.29	0.722
LAEDV (ml)	62.4±20.6	67.0±19.9	64.9±22.1	*F*(2 , 84)=1.34	0.267
LAESV (ml)	38.9±13.9	34.9±14.2	31.1±13.3	*F*(2 , 84)=10.47	<0.001
LAEF (%)	37.3±12.5	48.7±9.7	52.7±9.2	*F*(1.83, 76.8)=30.75	<0.001
LAεR (%)	11.9±7.0	20.5±5.2	25.6±6.8	*F*(2 , 84)=56.99	<0.001
LAεCD (%)	−7.7±5.0	−11.4±3.2	−13.2±4.0	*F*(1.73 , 72.75)=23.63	<0.001
LAεCT (%)	N/A*	−9.0±3.3	−12.1±4.5	*t*(42)=−2.4^§^	0.023
LVEDV (ml)	87.4±21.2	112.2±16.4	117.5±21.2	*F*(2 , 84)=55.97	<0.001
LVESV (ml)	39.2±11.2	49.3±12.6	46.3±10.6	*F*(2 , 84)=13.55	<0.001
LVEF (%)	55.9±8.4	56.2±7.4	60.5±6.4	*F*(1.79 , 75.11)=7.76	0.001

Figures are mean ± standard deviation.

*Non-applicable during atrial fibrillation

^§^Paired sample *t*-test

*ANOVA*, analysis of variance; *CV*, cardioversion; *LAEDV*, left atrial end-diastolic volume; *LAESV*, left atrial end-systolic volume; *LAEF*, left atrial total emptying fraction; *LAεR*, left atrial peak longitudinal strain during reservoir function; *LAεCD*, left atrial peak longitudinal strain during conduit function; *LAεCT*, left atrial peak longitudinal strain during contractile function; *LAVI*, left atrial volume index; *LVEDV*, left ventricular end-diastolic volume; *LVESV*, left ventricular end-systolic volume; *LVEF*, left ventricular ejection fraction; *ml*, milliliter; *m*, meter

**Fig. 2:**
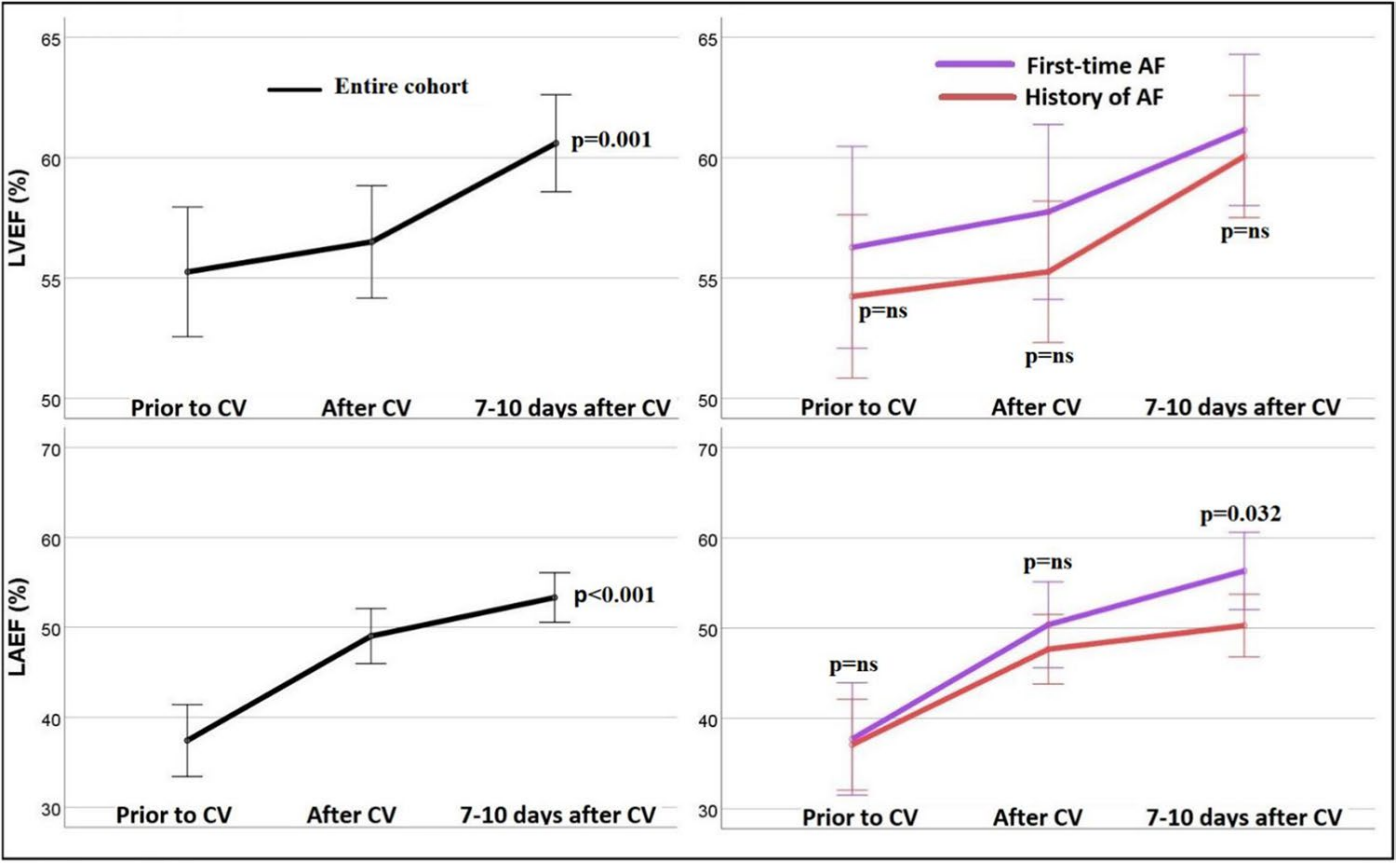
Left atrial and left ventricular ejection fraction. Graphs represent ordinary mean plots and interaction plots, error bars, 95% confidence interval; groups according to positive or negative atrial fibrillation history; AF: atrial fibrillation; CV: cardioversion; LAEF: left atrial emptying fraction; LVEF: left ventricular ejection fraction; Par.AF: paroxysmal atrial fibrillation; Per.AF: persistent atrial fibrillation.

Although in all study participants the LVEDV, LVESV, and LVEF were all within the normal ranges prior to CV, the LVEF further improved significantly after CV (Fig. 2). Significantly higher LVEDV was also observed after CV, as compared to prior to CV. Moreover, the increase in LVEDV was independent of the ventricular rate during AF, even when the cohort was dichotomized by the ventricular rate below or above 110 beats per minute.

The ICC for inter-rater reliability was between fair and good; detailed results are presented in *supplement table* *2*.

### Inflammatory, cardiac, and coagulative biomarkers

Amongst biomarkers of inflammation and endothelial dysfunction, IL-6 and P-selectin showed a statistically significant overall decrease in the serial measurement analysis from prior to CV to the 7–10 days after CV. The CRP did not change significantly. Both cardiac biomarkers, hs-cTNT and NTpro-BNP, decreased statistically significantly, after CV. The biomarkers of coagulation, PTf1+2 and fibrinogen, decreased statistically significantly in the serial analysis from prior to CV to the 7–10 days after CV. The vWfAg increased transiently after CV and decreased 7–10 days after CV, showing an overall statistically significant change in serial analysis. The FVIII and D-Dimer did not change significantly (Table 3). The levels of observed biomarkers did not differ in patients with versus without prior history of AF, or in patients spontaneously versus electrically cardioverted.

**Table 3:** Biomarker tests; pairwise comparison and serial measurements analysis

	Biomarker (Ref. units)	Prior to CV	Post CV	Prior versus post CV		7–10 days after CV	Post CV versus 7–10 days		Overall change	
				*Z* score^#^	*p* value^§^		*Z* score^#^	*p* value^§^	*χ*^2^ (2)^##^	*p* value
Inflammatory biomarkers	CRP (<5 mg/L)	1.0 (0.71–2.86)	1.0 (0.71–2.28)	−1.57	0.3	1.5 (0.71–3.0)	-0.83	0.8	3.22	0.2
	IL-6 (0.495–3.92 ng/L)	2.3 (0.95–2.48)	2.0 (1.1–2.72)	−0.86	1	1.7 (0.63–1.97)	3.82	<0.001	16.22	<0.001
	P-selectin (0.8–50 ng/mL)	36.7 (27.9–44.2)	34.3 (24.4–38.9)	2.31	0.061	34.9 (29.2–38.4)	0.53	1	9.27	0.01
Cardiac biomarkers	hs-cTNT (<14 ng/L)	6.0 (5.0–9.0)	6.0 (3.5–8.0)	3.72	0.001	5.1 (3.5–7.9)	1.46	0.4	32.46	<0.001
	NT pro-BNP (<330 ng/L)	1200 (827–1750)	882 (534–1360)	3.56	0.001	86 (45–135)	4.85	<0.001	71.30	<0.001
Coagulation biomarkers	PTf1+2 (69–229 pmol/L)	194 (134–204)	169 (113–199)	2.59	0.029	161 (110–185)	0.48	1	10.98	0.004
	vWfAg (0.60–1.60 kIE/L)	1.23 (0.99–1.7)	1.36 (0.99–1.64)	0.97	0.9	1.23 (0.9–1.42)	1.45	0.44	6.25	0.044
	FVIII (0.50–1.80 kIE/L)	1.44 (1.16–1.96)	1.44 (1.04–1.85)	−1.73	0.08	1.38 (1.03–1.8)	−0.89	0.6	3.28	0.19
	Fibrinogen ( 2.0–4.2 g/L)*	3.50 (3.15–4.37)	3.45 (3.07–4.05)	1.98	0.1	3.30 (3.17–4.45)	0.28	1	6.84	0.033
	D-dimer ( <900 ng/L)*	469 (275–993)	409 (249–1326)	−0.97	0.3	456 (281–1109)	0.59	0.5	2.71	0.25

^#^Wilcoxon signed-rank test used for pairwise comparison

^##^Friedman’s two-way analysis of variance by ranks used for analysis of overall change in serial measurements

*CRP*, C-reactive protein; *FVIII*, coagulation factor VIII; *hs-cTNT*, high-sensitivity cardiac troponin T; *IL-6*, interleukin-6; *NT-proBNP*, N-terminal pro-brain natriuretic peptide; *Post CV*, 1–3 h after cardioversion; *PTf1+2*, prothrombin fragment 1+2; *Ref*, reference interval; *vWFag*, von Willebrand Factor antigen

^**§**^Significance values adjusted by the Bonferroni correction for multiple tests

*Only in a subgroup of 14/43 patients

### Chronic white matter hyperintensities

**Fig. 3:**
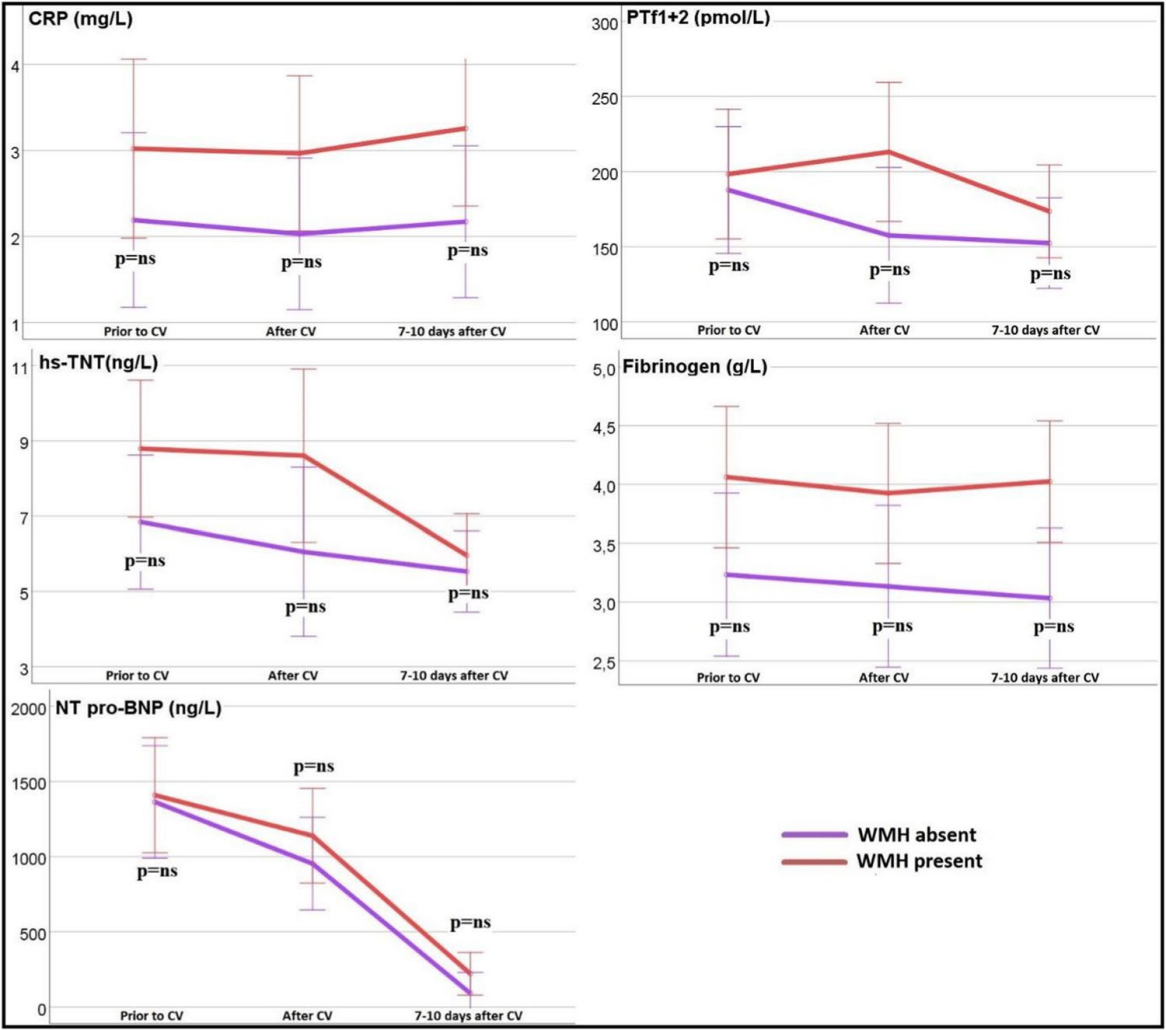
Biomarkers association with white matter hyperintensities. Graphs represent ordinary mean plots and interaction plots, error bars, 95% confidence interval; CRP: C-reactive protein; hs-cTNT: high-sensitivity cardiac troponin-T; NT-proBNP: N-terminal pro-brain natriuretic peptide; PTf1+2: prothrombin fragment 1+2; WMH: white matter hyperintensities.

Brain MRI prior to CV showed chronic white mater lesions or white matter T2 hyperintense lesions in 21/43 (49%) of the patients, of whom 18/43 (42%) had Fazekas score 1 and 3 (7%) Fazekas score 2. Patients with WMH showed a tendency of higher CRP, hs-cTNT, NTpro-BNP, and PTf1+2 as compared with patients without WMH, but the difference did not reach statistical significance (Fig. 3). No association was found between the presence of previous AF history and presence of WMH. No patient had signs of recent acute ischemic lesions.

## Discussion

In this prospective observational study with serial echocardiographic examinations for the evaluation LA stunning in conjunction with blood sampling of various biomarkers reflecting inflammatory, coagulative, and cardiac changes peri- and post-cardioversion, we were able to detect signs of enhanced thrombogenicity, even in low-risk patients with recent onset AF. The inclusion of only anticoagulant-naïve patients eliminated interactions related to the coagulative system making interpretations easier.

Abnormal blood flow, the first element of Virchow’s triad, corresponds to the left atrial hemodynamics. Left atrial mechanical stunning, after CV when sinus rhythm was restored, was evident by the decreased LAEF, which normalized within 10 days. Despite the significant LA functional recovery and LAEF normalization at 7–10 days after restoration of sinus rhythm, the LAEF was still significantly lower in patients with a history of paroxysmal or persistent AF compared with those with new-onset AF. A plausible explanation for the lower LAEF in patients with a prior AF history is a slower reverse remodeling which may be secondary to irreversible structural remodeling after their previous episodes of AF. These findings are consistent with another study reporting lower LAEF in patients with persistent or permanent AF as compared with paroxysmal AF and increased LAVI with the severity of AF type [21]. Moreover, LAEF seems to be significantly lower in persistent AF patients with arrhythmia recurrences after LA ablation versus those without, indicating that more extensive irreversible remodeling is an arrhythmia precursor [22].

In the present study, the LA peak longitudinal strain during reservoir and conduit phase prior to CV were lower in patients with previous AF history as compared to those without. Although LAεR, LAεCD, and LAεCT improved significantly, they failed to normalize in both of these patient groups at 7–10 days. The presence of irreversible remodeling and fibrosis, identified during electro-anatomical mapping as low-voltage zones, have in other studies been associated with decreased LAEF, LAεR, LAεCD, and LAεCT in paroxysmal AF patients [23]. Moreover, extensive LA late gadolinium enhancement (LGE) on cardiac MRI, reflecting fibrosis, was inversely associated with LA emptying fraction, longitudinal strain during reservoir, conduit, and contractile phases; the latter assessed with cardiac MRI feature-tracking corresponding to STE analysis [24]. Left atrial deformation indices are strongly associated with LA fibrosis and AF progression, and as decreased indices are observed in patients with cryptogenic stroke, these early signs of LA remodeling may be a used to identify or predict a thrombogenic milieu [23–26].

Endothelial/tissue dysfunction and hypercoagulability, the remaining two elements of Virchow’s triad, correspond to inflammatory/cardiac biomarkers and coagulation biomarkers respectively. The observation of the significant overall decrease of IL-6, P-selectin, hs-cTNT, NTpro-BNP, PTf1+2, and fibrinogen, and the significant transient increase of vWfAg, from the time while in AF to the time of reverse remodeling after restoration of sinus rhythm is consistent with previous reports [2, 27]. Elevated levels of IL-6 and CRP, indicating inflammatory activity, were not only associated with AF severity, but were also identified as prognostic markers for AF recurrence after CV and catheter ablation, as well as for stroke.[2, 27] Elevated CRP and IL-6 levels have previously been associated with increased LA size and long-lasting AF episodes indicating an association to LA structural changes and remodeling [28]. The maintenance of sinus rhythm after cardioversion resulted in a gradual decrease of CRP suggesting that inflammation is a consequence of AF [29].

Higher troponin levels and elevated NTpro-BNP have been associated with incident AF, with AF recurrence after CV, and with thromboembolic risk [30–34]. A sustained increase in troponin levels after CV has also been associated with AF recurrence, although the pathophysiologic mechanism is not clearly understood [30, 35]. A hypercoagulability state in AF, as was reflected by elevated PTf1+2, fibrinogen, and vWfAg in the present study, has previously been reported even during the first 24 h of paroxysmal AF, observations that are in line with the current study [36–40].

Moreover, in the present study, a non-significant trend for higher CRP, hs-cTNT, NTpro-BNP, and PTf1+2 values was found in patients with brain WMH. Although CRP and Pf1+2 have been associated with the presence and severity of WMH in the elderly, and hs-cTNT and fibrinogen with the presence of WMH in patients with acute ischemic stroke, little is known about their relation to WMH in the AF population without stroke [41–47]. Since AF is associated not only to the presence of WMH, but also to the activation of inflammatory and coagulation systems, the findings in the present study further support the perception that AF is a marker of a cardiovascular disease, linking AF and WMH, beyond thromboembolism [13, 14, 48, 49].

### Limitations

The major limitation in the present study is the small size of the cohort and the underrepresentation of women. Moreover, left atrial volumes were measured only using the 4-chamber view and left ventricular volumes were measured as a mean of three consecutive beats during AF. Finally, the observed changes in inflammatory and coagulation biomarkers, although significant, were very small, underlining that the analysis of circulating biomarker should be interpreted with caution and used as hypothesis generating.

## Conclusion

The observation of left atrial mechanical stunning followed by reverse functional remodeling in conjunction with the transiently higher level of inflammatory and coagulative biomarkers during AF might support the presence of an enhanced thrombogenicity even in patients with an inherent low risk for stroke. The finding of a more pronounced mechanical stunning in patients with a prior history of atrial fibrillation further supports the concept of adequate anticoagulation pericardioversion even in low-risk patients.

### Author contribution

Panagiotis Arvanitis: conceptualization, methodology, formal analysis, investigation, data curation, writing - original draft, writing—review and editing, visualization, project administration. Anna-Karin Johansson: investigation, data curation, writing—review and editing. Mats Frick: investigation, resources, writing—review and editing. Helena Malmborg: investigation, writing—review and editing. Spyridon Gerovasileiou: investigation: echocardiography, writing—review and editing. Elna-Marie Larsson: investigation: brain magnetic resonance imaging, writing—review and editing. Carina Blomström-Lundqvist: conceptualization, methodology, ethics application, formal analysis, investigation, resources, writing—review and editing, supervision, project administration, funding acquisition.

## Supplementary Information

ESM 1 (DOCX 37 kb)
