# Supplementary material for: Recent-onset atrial fibrillation: a study exploring the elements of Virchow’s triad after cardioversion
Source: J Interv Card Electrophysiol. 2021 Oct 24;64(1):49–58. doi: 10.1007/s10840-021-01078-9 (PMC9236986; doi:10.1007/s10840-021-01078-9)
Supplement: Supplementary file 1 — (DOCX 37 kb) [file 10840_2021_1078_MOESM1_ESM.docx]

## Supplementary material

### Supplement table 1. Inflammatory, coagulation and cardiac biomarkers: measurement method and reference interval

| **Biomarker** | **Measurement method** | **Reference interval** |
| --- | --- | --- |
| CRP | CRPhs, Roche Diagnostics | <5 mg/L |
| IL-6 | Quantikine ® HS Human IL-6 Immunoassay, ELISA, R&D Systems | 0.495 – 3.92 ng/L |
| P-Selectin | Quantikine ®Human P-Selectin/CD62P, ELISA, R&D Systems | 0.8 - 50 ng/mL |
| hs-cTNT | Elecsys® hs-Troponin T, Roche Diagnostics | <14 ng/L |
| NT-proBNP | ARCHITECT i2000 BNP (Abbott, Abbott Park, Illinois) | <330 ng/L |
| PTf1+2 | Enzygnost® F 1+2 (monoclonal), Siemens | 69 – 229 pmol/L |
| vWFag | STA®-Liatest® VWF:Ag, STA-R Max, Stago | 0,60 – 1,60 kIE/L |
| F-VIII | Asserachrom® VIII:Ag STA-R Max, Stago | 0,50 – 1,80 kIE/L |
| Fibrinogen | STA®-Fibrinogen 5, STA-R Max, Stago | 2,0–4,2 g/L |
| D-Dimer | STA®-Liatest® D-Di, STA-R Max, Stago | <900 ng/L |

CRP: C-reactive protein; F-VIII: Coagulation factor VIII; hs-cTNT: high-sensitivity cardiac troponin T; IL-6: Interleukin-6; NT-proBNP: N-terminal pro-brain natriuretic peptide; PTf1+2: Prothrombin fragment 1+2, vWFag: von Willebrand factor antigen;.

### Supplement table 2. Interrater agreement analysis for echocardiographic results

| **Echocardiographic parameter** | **Rater 1, (mean)** | **Rater 2, (mean)** | **ICC** | **95% CI** |
| --- | --- | --- | --- | --- |
| LAEDV (ml) | 53 | 46 | 0,697 | 0,263 – 0,860 |
| LAESV (ml) | 29 | 25 | 0,782 | 0,529 -0,893 |
| LAEF (%) | 45 | 46 | 0,650 | 0,334 – 0.815 |
| LAεR (%) | 18 | 18 | 0,799 | 0,617 – 0,894 |
| LAεCD (%) | -8 | -9 | 0,587 | 0,216 – 0,782 |
| LAεCT (%) | -10 | -12 | 0,716 | 0,176 – 0,902 |
| LVEDV (ml) | 109 | 104 | 0,864 | 0,737 - 0,929 |
| LVESV (ml) | 47 | 43 | 0,827 | 0,615 – 0,916 |
| LVEF (%) | 56 | 58 | 0,813 | 0,640 – 0,902 |

ICC: Intraclass correlation coefficient; Values less than 0.5 are indicative of poor agreement, values between 0.5 and 0.75 indicate fair agreement, values between 0.75 and 0.9 indicate good agreement, and values greater than 0.90 indicate excellent agreement; CI: Confidence interval; LAEDV: Left atrial end diastolic volume; LAESV: Left atrial end systolic volume; LAEF: Left atrial emptying fraction; LAεR: Left atrial peak longitudinal strain during reservoir function; LAεCD: Left atrial peak longitudinal strain during conduit function; LAεCT: Left atrial peak longitudinal strain during contractile function; LVEDV: Left ventricular end diastolic volume; LVESV: Left ventricular end systolic volume; LVEF: Left ventricular ejection fraction.
